# Combining Immune Checkpoint Inhibitors: Established and Emerging Targets and Strategies to Improve Outcomes in Melanoma

**DOI:** 10.3389/fimmu.2019.00453

**Published:** 2019-03-19

**Authors:** Duaa O. Khair, Heather J. Bax, Silvia Mele, Silvia Crescioli, Giulia Pellizzari, Atousa Khiabany, Mano Nakamura, Robert J. Harris, Elise French, Ricarda M. Hoffmann, Iwan P. Williams, Anthony Cheung, Benjamin Thair, Charlie T. Beales, Emma Touizer, Adrian W. Signell, Nahrin L. Tasnova, James F. Spicer, Debra H. Josephs, Jenny L. Geh, Alastair MacKenzie Ross, Ciaran Healy, Sophie Papa, Katie E. Lacy, Sophia N. Karagiannis

**Affiliations:** ^1^St. John's Institute of Dermatology, School of Basic & Medical Biosciences, Guy's Hospital, King's College London, London, United Kingdom; ^2^School of Cancer & Pharmaceutical Sciences, Guy's Hospital, King's College London, London, United Kingdom; ^3^Breast Cancer Now Research Unit, School of Cancer & Pharmaceutical Sciences, Guy's Cancer Centre, King's College London, London, United Kingdom; ^4^Department of Plastic Surgery at Guy's, King's, and St. Thomas' Hospitals, London, United Kingdom

**Keywords:** checkpoint inhibitors, combination immunotherapy, immunooncology therapeutics, melanoma, CTLA-4, PD-1, PD-L1, antibody engineering

## Abstract

The immune system employs several checkpoint pathways to regulate responses, maintain homeostasis and prevent self-reactivity and autoimmunity. Tumor cells can hijack these protective mechanisms to enable immune escape, cancer survival and proliferation. Blocking antibodies, designed to interfere with checkpoint molecules CTLA-4 and PD-1/PD-L1 and counteract these immune suppressive mechanisms, have shown significant success in promoting immune responses against cancer and can result in tumor regression in many patients. While inhibitors to CTLA-4 and the PD-1/PD-L1 axis are well-established for the clinical management of melanoma, many patients do not respond or develop resistance to these interventions. Concerted efforts have focused on combinations of approved therapies aiming to further augment positive outcomes and survival. While CTLA-4 and PD-1 are the most-extensively researched targets, results from pre-clinical studies and clinical trials indicate that novel agents, specific for checkpoints such as A2AR, LAG-3, IDO and others, may further contribute to the improvement of patient outcomes, most likely in combinations with anti-CTLA-4 or anti-PD-1 blockade. This review discusses the rationale for, and results to date of, the development of inhibitory immune checkpoint blockade combination therapies in melanoma. The clinical potential of new pipeline therapeutics, and possible future therapy design and directions that hold promise to significantly improve clinical prognosis compared with monotherapy, are discussed.

## Introduction

Immune-mediated destruction of tumors has long been considered a potential route of therapeutic intervention. Partial spontaneous regression of melanoma lesions has previously been associated with the presence of endogenous tumor infiltrating lymphocytes (TILs) and the presence of TILs in patient samples has been shown to correlate with improved clinical outcomes and better prognosis (1). Infusion with CD8+ TILs has been reported to induce some responses in patients when combined with other treatments including IL-2 (2). Immunotherapy via cytokine infusion has also been extensively trialed, with IL-2, IL-12, and IFNα2b to activate T cells, showing anti-tumor effects in pre-clinical models and clinical trials, with IL-2 and IFNα2b approved for clinical use (3, 4). Cytokine treatments have however been associated with severe adverse effects resembling severe systemic infections and sometimes resulting in toxic shock or capillary leak syndrome as reported in randomized clinical trials (5, 6). Though not without challenges, these trials confirmed the possibility of reigniting components of the immune system as a cancer therapy.

**Figure 1:**
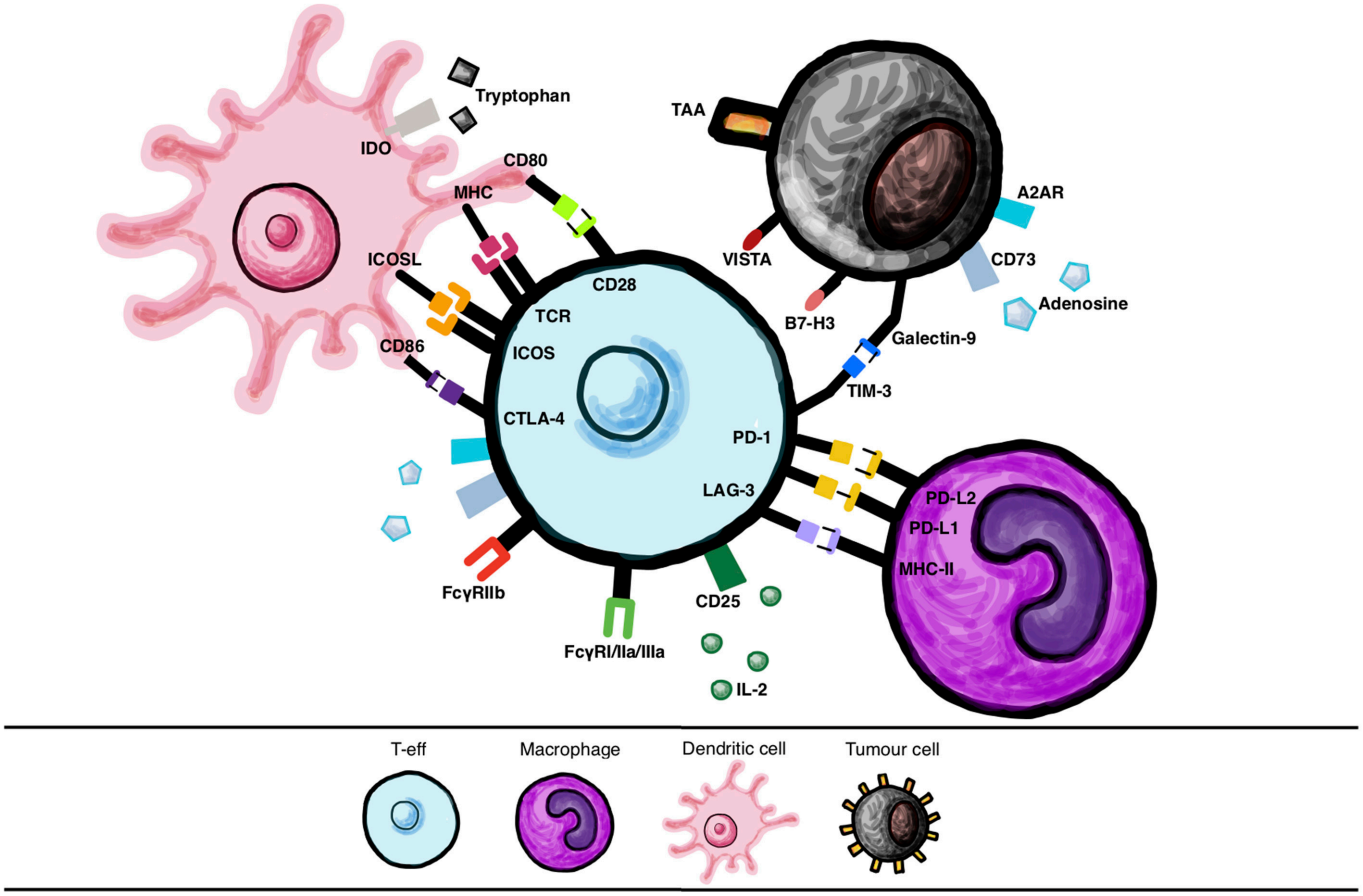
Immune cell interactions via checkpoint molecules and their ligands. Various interactions between checkpoint molecules and their ligands expressed by different cells, such as immune cells (dendritic cells (DC)s, T-effector cells (T-eff), macrophages) and between T-eff and tumor cells, that may be targeted with therapy.

Increased understanding of tumor evolution and the complex interactions in the tumor microenvironment (TME) has revealed numerous mechanisms by which tumors may escape immune destruction and actively suppress immune activity (7). Immunosuppression by tumor cells may partially be mediated through FoxP3+ regulatory T cell (T-reg) recruitment via tumor-secreted chemokines as shown in an *ex vivo* study (8, 9). Critically, tumor resident T-reg can highly express cytotoxic T-lymphocyte-associated antigen-4 (CTLA-4), an important checkpoint that acts as a negative regulator of effector T cell (T-eff) activity *in vivo*, studied in different models including CTLA-4-deficient mice (10) (Figure 1). Suppression may also be mediated by tumor expression of the Programmed-death ligand 1 (PD-L1; B7-H1; CD274), known to trigger T cell apoptosis *in vivo* in mouse tumors (11) and to promote formation of FoxP3+ T-regs upon interaction with the T cell-associated checkpoint receptor Programmed-death 1 (PD-1, also known as CD279) (12) (Figure 1). These checkpoints, have become therapeutic targets in immune checkpoint blockade therapy, with the aim of overcoming TME-mediated immunosuppression and restoring anti-tumor immune activity (13). Monoclonal antibodies targeting CTLA-4 and PD-1 have now been approved for the treatment of melanoma. These new therapeutic modalities were developed in parallel with targeted MAPK pathway inhibitor therapies, such as vemurafenib and dabrafenib, approved for a subset of melanomas bearing point mutations in the kinase BRAF (e.g., BRAF^V600E^), and the MEK inhibitors trametinib and cobimetinib, all designed to cause cancer cell death via interruption of the MAPK pathway (Table 1). Together, these agents have led to an increase in medial survival for advanced melanoma from 9 months in 2010 to over 3.5 years.

**Table 1:** Approved targeted, antibody and other immunotherapies and combination treatments for malignant melanoma.

**Drug**	**Target**	**Mechanism**	**Indication**	**Approval**
**MONOCLONAL ANTIBODY MONOTHERAPIES**				
Ipilimumab	CTLA-4	Human IgG1 monoclonal antibody blockade	Advanced unresectable metastatic melanoma	2011
Nivolumab	PD-1	Human IgG4 monoclonal antibody blockade	Advanced metastatic melanoma ± refractory to ipilimumab	2014^*^
				^*^2017: Approved as adjuvant treatment for melanoma with involvement of lymph nodes or for patients with metastatic disease who have undergone complete resection
Pembrolizumab	PD-1	Humanized IgG4 monoclonal antibody blockade	Unresectable melanoma—stage III/IV	2014
**COMBINED MONOCLONAL ANTIBODY THERAPIES**				
Ipilimumab + nivolumab	CTLA-4 + PD-1	Monoclonal antibody blockade	Unresectable melanoma—stage III/IV PD-L1 negative	2015
**TYROSINE KINASE INHIBITOR MONOTHERAPIES**				
Vemurafenib	BRAF	BRAF inhibitor causing programmed cell death via interruption of MAPK pathway	Unresectable BRAF^V600^ mutant melanoma	2011
Dabrafenib	BRAF inhibitor	BRAF inhibitor causing programmed cell death via interruption of MAPK pathway	Unresectable BRAF^V600^ mutant melanoma (not wild-type)	2013
Trametinib	MEK inhibitor	MEK1 and 2 inhibitor causing cell death via interruption of MAPK pathway	Unresectable BRAF^V600E/K^ mutant melanoma (not to be used post BRAF inhibitor)	2013
**COMBINED TYROSINE KINASE INHIBITOR THERAPIES**				
Dabrafenib + trametinib	BRAF + MEK	BRAF+MEK inhibition	Unresectable BRAF^V600E/K^ mutant melanoma	2013
Vemurafenib + cobimetinib	BRAF + MEK	BRAF+MEK inhibition	BRAF^V600^ mutant melanoma	2015^**^
				^**^2018: Approved as adjuvant treatment for patients with nodal involvement and following complete resection
**OTHER TARGETED AND IMMUNE THERAPIES**				
Interferon	IFNα2b	Systemic IFNα2b administration results in immunostimulatory effects including an increase in tumor-infiltration, decrease in circulating T-regs and modulation of STAT1/STAT3 balance	Adjuvant therapy for stage III melanoma (cancer free but at high risk of recurrence) Adjuvant therapy for stage IIB or IIC melanoma with primary lesions >4mm thickness	1995
Aldesleukin	IL-2	Systemic IL-2 administration promotes T cell proliferation and stimulates CD8 and NK cell cytotoxicity	Metastatic melanoma	1998
T-VEC	Oncolytic herpes simplex virus	Local and direct infection and killing of tumor cells	Unresectable stage IIIB, IIIC or IV melanoma	2015

While CTLA-4 and PD-1 blockade has proved successful in improving survival rates, many patients do not respond or develop resistance to these interventions. Alongside combinations of checkpoint inhibitors already in clinical use, research into new checkpoints as therapeutic targets has shown promise in pre-clinical and clinical studies, either alone or combined with established agents. Focusing on malignant melanoma as the tumor type for which the first pivotal immunotherapy breakthroughs were demonstrated, in this review, we discuss current and future checkpoint blockade and other immunooncology combination therapies, and the rationale for potential synergistic effects (Table 2).

**Table 2:** Proposed mechanisms of action of selected immune checkpoint blocking agents.

**Agent**	**Specificity**	**Mechanism**
Ipilimumab	CTLA-4	• Inhibits coinhibitory checkpoint molecule CTLA-4 on T cells by preventing CD80/CD86 binding on APCs • Enhances T-eff activation and T-reg inhibition
Pembrolizumab/nivolumab	PD-1	• Inhibit coinhibitory checkpoint molecule PD-1 on antigen-educated T cells, preventing PD-L1-APC binding • Enhances T-eff activation • Reduces T-eff anergy
BMS-956559/PDR001	PD-L1	• Inhibits coinhibitory checkpoint molecule PD-L1 on APCs and melanoma cells preventing its binding to PD-1 and CD80/CD86 on T cells • Enhances T-eff activation • Reduces T-eff anergy
LAG525	LAG-3	• Inhibits coinhibitory checkpoint molecule LAG-2 on activated T cells preventing binding to MHC-II on DCs, pDC and melanoma cells • Enhances T-eff and myeloid cell responses (DCs, macrophages and NK cells
CP1-444	A2AR	• Inhibits coinhibitory checkpoint molecule A2AR molecule on myeloid cells preventing binding to extracellular adenosine released by CD73 on T-reg and melanoma cells • Enhances myeloid cell response • Reduces T-reg cell response
MBG453	TIM-3	• Inhibits coinhibitory checkpoint molecule TIM-3 on T cells preventing to binding on galactin-9 on immune and melanoma cells and HMG-B on immune cells • Prevents T cell exhaustion • Enhances nucleic acid recognition within endosomes
MGA271	B7-H3	• Inhibits coinhibitory checkpoint molecule B7-H3 on APCs and melanoma cells preventing ligand binding on T cells • Enhances T-eff activation and T-reg inhibition
CA-170	VISTA	• Inhibits coinhibitory checkpoint molecule VISTA on myeloid cells and naïve T cells preventing binding to VSIG-3 • Enhances T-eff activation and T-reg inhibition
Epacadostat	IDO	• Inhibits coinhibitory checkpoint molecule IDO on alternatively activated macrophages, T-regs and melanoma cells preventing the conversion of tryptophan to kynurenines on T cells • Enhances NK/ T-eff activation and T-reg inhibition
N/A	PKC-η	• Inhibits coinhibitory signaling molecule PKC-η on T-regs preventing its induction of anti-inflammatory cytokine transcription • Enhances T-eff activation and T-reg inhibition

## Therapies Targeting CTLA-4 and PD-1

### Anti-CTLA-4 Monotherapy

CTLA-4 is a CD28 homolog expressed constitutively on the surface of both T-reg cells and activated T cells (14). CTLA-4 binds to CD28 co-receptors CD80/60 with a higher affinity and avidity than CD28, thus superseding positive CD28 signaling and thus allowing for inhibition of T cell activation (15, 16). In order to function effectively as an immune checkpoint via endocytosis CTLA-4 is not only able to competitively inhibit T cell co-stimulation but can also clear CD28 ligands CD80/CD86 from the surrounding cells including APCs by trans-endocytosis *in vivo* (17). Physiologically, CTLA-4 has been shown *in vitro* and in mouse models *in vivo*, to suppress T cell responses including activation, proliferation, and pro-inflammatory cytokine production (IFN-γ and IL-2) by antigen-presenting cells (APCs) such as dendritic cells (DCs) and macrophages (18) (Figure 1).

Studies on cells expressing human CTLA-4 in murine models of melanoma, that investigated antibodies aimed at blocking CTLA-4 checkpoints, have documented effects such as enhanced T-eff function, inhibition of T-reg activity and selective depletion of T-reg cells via antibody Fc binding of Fcγ-receptors on atypical macrophages in tumor lesions (19, 20). Hypotheses that CTLA-4 blockade could enhance anti-tumor response were tested by a pre- clinical study using transplantable murine melanoma cell lines, demonstrating that CTLA-4 inhibitors induced rejection of melanoma (21). *Ex vivo* studies of peripheral blood mononuclear cells (PBMCs) and matched melanoma metastases from patients with melanoma treated with ipilimumab have shown evidence that ipilimumab also works by depleting T-reg cell populations by antibody-dependent cell-mediated cytotoxicity (ADCC) mediated by CD16 (FcγRIIIA)-expressing, nonclassical monocytes. In the same study, patients who responded to ipilimumab treatment had higher ratios of intratumoral CD68-expressing vs. CD163-expressing macrophages before treatment and lower T-reg infiltration after treatment (22). Clinical trials involving ipilimumab have demonstrated a dose-dependent response to the antibody in late-stage melanoma patients, with pooled analysis consistently showing improved survival in patients with metastatic disease above historical controls (23, 24). By blocking this key immune escape mechanism, overall survival rates for ipilimumab were significantly improved, alone or in combination with a glycoprotein 100 peptide (GP-100) vaccine when compared to vaccine alone (15, 25). Ipilimumab, a fully humanized IgG1 antibody, was the first anti-CTLA-4 treatment approved by FDA in 2011 (Table 1).

### Anti-PD-1 Monotherapy

Another immune checkpoint, the programmed death 1 (PD-1) immunoglobulin-based receptor predominantly expressed on activated, antigen-educated T cells can recognize two ligands, PD-L1 and PD–L2 (B7-DC; CD273). PD-L1 is expressed broadly across many cell types, including leukocytes and tissue cells, whereas PD-L2 expression is limited and specific to expression on immune cells: antigen presenting and stromal cells. Ligation of PD-1 to PD-L1 causes phosphorylation and activation of SHP-2, a phosphatase that can inactivate many downstream molecules in TCR signaling (26). *In vitro* and *in vivo* studies in mouse models of cancer showed that PD-L1 can also enhance the generation of peripherally induced T-regs, (iT-reg), increasing Foxp3 expression and sustaining their immunoregulatory actions such as suppression of CD4^+^ T-eff cells (27). The co-stimulatory molecule CD28 of which CTLA-4 is a homolog, is also preferentially targeted by PD-1-mediated dephosphorylation (28). By this mechanism, PD-1 mediates two immune checkpoints, by reducing immune hyperstimulation via PD-L1 and maintaining tolerance in lymphoid tissues via PD-L2. Both ligands PD-L1 and PD-L2 can also be induced by cytokine signaling during inflammation (29).

PD-L1 expression on tumor cells is often upregulated, resulting in inhibition of T cell responses (15). In melanoma, the expression of PD-L1 may be prognostic, and could correlate with Breslow thickness (30). Mouse melanoma metastasis to the liver was shown to be impaired in PD-1-deficient mice and anti-PD-1 monoclonal antibody administration could inhibit the spread of tumor cells via recruitment of T-eff (31) by blocking the interaction of PD-1 with its ligands (14). Anti-PD-1 and anti-PD-L1 blocking strategies produce different immunologic effects as anti-PD-L1 has effects on more than one pathway. PD-L1 signals negatively to T cells by interacting with both CD80/CD86 and PD-1 (32) preventing both pathways, without interacting with PD-L2, which activates T cell response by producing co-stimulatory signals (32). Anti-PD-L1 studies demonstrated temporary arrest of the growth of melanoma cells in mouse models (33). Following the approval of ipilimumab, the anti-PD-1 monoclonal antibodies nivolumab and pembrolizumab gained FDA approval in 2014 (34) and EMA approval in 2015, following trials showing significantly improved patient outcomes (Table 1) (35). Nivolumab is a fully human IgG4 monoclonal antibody which was shown to improve median overall survival to 8.9 months compared to 6.8 months in patients treated with dacarbazine in a phase III study involving patients with previously untreated melanoma (36). Studies into metastatic melanoma have shown superior overall survival of 1 year (72.9 to 42.1%) and better objective response rate (40 to 13.9%) with nivolumab plus dacarbazine compared to dacarbazine plus placebo (36). Pembrolizumab, a humanized IgG4 monoclonal antibody has also shown similar efficacy to nivolumab, with one phase III study reporting increased long-term survival rates when compared to ipilimumab in patients with unresectable melanoma (37).

### Comparing Anti-CTLA-4 and Anti-PD-1 Therapies and Toxicity in the Clinic

Both anti-CTLA-4 and anti-PD-1 therapies aim to restore T cell effector function in the TME and establish immune dominance over tumors. Overall, these immune checkpoint blockade monotherapies have generated significant improvements in patient outcomes against traditional dacarbazine therapy, with anti-PD-1 blockade seemingly more effective.

In 173 patients with advanced melanoma unresponsive to ipilimumab treatment, the overall response rate (ORR) to pembrolizumab was 26%, with the most severe adverse event (AE) reported as grade 3 fatigue in 5 patients (38). In comparison, CTLA-4-blockade treatment-associated toxicity has not been insignificant; 60% of patients treated with ipilimumab experienced adverse immune effects, which were severe (grade 3 or 4) in 10–15% of cases (25). Head-to-head trials of pembrolizumab vs. ipilimumab have shown 47.3 and 26.5% 6-month progression-free-survival (PFS) rates respectively, with AEs grade 3 or higher at rates of 19.9 and 13.3% (39). Nivolumab was shown to be effective in a range of cancers, producing a 28% ORR in melanoma and grade 3 or 4 drug- related AEs occurred in 14% of 296 patients across all groups (including three deaths from pulmonary toxicity) (40). Research in one study demonstrated 1-year and 2-year survival rates of 62 and 43%, respectively (35). Although both therapies have been successful in treating many cases, anti-PD-1 have thus far been more efficacious than anti-CTLA-4 monoclonal antibodies and have fewer adverse drug reactions. This difference may be because PD-1 is expressed on mature T cells; PD-L1 is expressed on antigen-presenting cells such as DCs and macrophages, and other immune cells as well as on tumor cells, while CTLA-4 is widely expressed on T cells across the body including those circulating in lymph nodes and skin. CTLA-4 inhibitor-mediated anti-tumor activity may therefore extend to secondary lymphoid organs rather than only within the TME (13). This wide expression distribution may potentially result in the disruption of other immune-regulating mechanisms and triggering of autoimmune-like events, consistent with toxicities observed in the clinic with anti-CTLA-4 antibody treatment.

### Rationale and Pre-clinical Evidence for Checkpoint Blockade Combination Therapy

While checkpoint inhibitor monotherapy provides significant benefits, this is generally only the case in subsets of patients. CTLA-4 and PD-1 are not functionally redundant, acting at different locations and times in the generation of T-eff (41). This may mean that combination therapies may act in a complementary or even synergistic fashion.

Checkpoint blockade therapies are also known to be subject to various forms of resistance mechanisms. Anti-CTLA-4 blockade primary resistance has been shown to correlate with a loss of IFN-γ signaling genes *in vitro* and clinically in patients who had poor clinical responses to ipilimumab therapy (42). Furthermore, in patients, PD-L1 expression on circulating CD4+ T CD8+ T cells may be predictive of resistance to anti-CTLA-4 treatment, providing a potential rationale for combination with PD-1/PD-L1 blockade therapy (43). Studies in murine models and patients receiving anti-PD-1 treatment also point to key roles of infiltrating myeloid cells and their signaling pathways, as well as upregulation of alternative immune checkpoints such as T cell immunoglobulin mucin-3 (TIM-3), all associated with resistance to checkpoint inhibition (44, 45). Checkpoint inhibitor monotherapy has been shown to trigger activation of compensatory T cell-associated checkpoints. Pre-clinical evidence supports a rationale for combination therapy as, by blocking more than one of these pathways, including PD-1, LAG-3, and CTLA-4, may reduce tumor growth (46, 47). Murine studies demonstrated that B16 melanoma cell rejection in mice was improved by combined anti-CTLA-4 and anti-PD-1 antibody therapies (47). The results indicated that combination therapy was more than twice as effective as monotherapy in terms of B16 melanoma rejection by increasing T cell infiltration and the presence of T-eff in the TME; IFN-γ and other pro-inflammatory cytokines were observed to be upregulated, producing an inflammatory rather than immunosuppressive TME (47). Further pre-clinical studies suggested that anti-CTLA-4 and anti-PD-1 therapies may have synergistic effects, increasing the numbers of TILs, reducing T-reg and retarding tumor growth (48). Certain subgroups of patients, such as elderly patients, tend to respond better to anti-PD-1 agents, a phenomenon which may be attributed to a depletion of the number of T-regs in older patients (49). These findings, taken together with studies suggesting that anti-CTLA-4 treatment can reduce CTLA-4-expressing T-regs, may lend further merit to stratifying appropriate patient groups to receive combination therapies (20, 22).

Therefore, since CTLA-4 and PD-1 inhibitors exert anti-tumor effects through different mechanisms of action, combining these agents could potentially lead to more efficacious treatments. Furthermore, blockade of one pathway resulted in increased activity and an upregulation of other inhibitory pathways, an effect that could perhaps be mitigated by combined therapy. Emerging evidence provided a rationale for the development of combined blockade regimens as well as acceleration of research into further blockade targets. Although toxicity profiles especially those associated with CTLA-4 blockade use may be an important concern in combined therapies, pre-clinical and clinical findings into the efficacy of combining checkpoint blockade antibodies showed encouraging results in melanoma.

### Combined CTLA-4 and PD-1 mAb Therapies in the Clinic

In the first clinical trial investigating the efficacy and safety of combined checkpoint blockade antibodies published in 2013 (50), 53 patients with melanoma were treated concurrently with nivolumab and ipilimumab, while 33 patients received only ipilimumab. In the double therapy group the ORR was 40% for combination treatment and the ORR was 20% in the monotherapy group. However, drug-related toxicity was higher in the combined therapy group; 53% of patients experienced grade 3 or 4 AEs compared to 18% in the monotherapy group. These drug related reactions were managed with medications.

Prolonged PFS was also reported in a phase II dose-escalation study of combined nivolumab and ipilimumab in 142 patients (51). ORRs in the combination and ipilimumab alone groups were 61 and 11%, respectively with drug-related AEs of grade 3 or 4 were exhibited by 54 and 24% of the patients, respectively (51). These drug-related AEs were also managed by immune modulation drug intervention. Follow up on these patients (median 24.5 months) showed a 63.8% 2-year overall survival for the combination group compared to 53.6% for ipilimumab alone (51). This clearly indicated the benefit of combined therapy and its longevity.

The registration phase III Checkmate 067 trial randomized 945 previously-untreated patients to nivolumab, ipilimumab and the two combined drugs, showing a median PFS of 6.9 months, 2.9 months and 11.5 months and 3-year overall survival (OS) rates of 52, 34, and 58%, respectively (52). This large well powered trial confirmed the superior efficacy of combination therapy and nivolumab monotherapy when compared to ipilimumab monotherapy as PFS was consistently longer for patients taking preparations that included nivolumab; this included subgroups categorized by PD-L1 or BRAF mutation status and metastasis stage (53). For patients with BRAF mutations the PFS was 11.7 months (11.2 months for those with wild-type BRAF). Positive PD-L1 status patients fared better with a median PFS of 14 months in both the nivolumab mono and dual therapy groups compared to only 3.9 months for patients taking ipilimumab alone. Checkmate 067 also reported higher rates of complete response in patients on a combined regimen (11.5% compared with 8.9% for nivolumab alone and only 2.2% for ipilimumab alone). Tumor burden change (a parameter which can be used for predicting treatment response) was also significantly higher in the combination group;−51.9% compared with−34.5% and 5.9% for nivolumab alone and ipilimumab alone, respectively.

Furthermore, treatment-related adverse events, as reported in the Checkmate 067 trial comparing ipilimumab, nivolumab and the two combined, revealed higher rates of toxicity associated with the combination therapy (96% compared with 86% in both monotherapy regimens) and this also more frequently led to discontinuation of treatment in the combined group than either monotherapy group. Grade 3 or 4 AEs occurred in 59% of patients given dual therapy compared with 21 and 28% of patients on nivolumab or ipilimumab alone, respectively, and these grade 3 or 4 adverse reactions were most frequently gastrointestinal in nature. Side effects were managed with established safety guidelines and usually resolved within 3–4 weeks and the 3-year survival rate for patients who discontinued treatment was 67% (52). These indicate that combined treatment elicited higher rates of toxicity than either monotherapy and that benefit from dual therapy was conferred despite discontinuation of treatment. Median survival in patients with PD-L1-positive tumors was the same in both the combination and nivolumab alone groups. This may reflect T cell infiltration enhanced by ipilimumab, thus favoring a TME that may be amenable to anti-PD-1 agent action (54).

Previous studies of anti-PD-1 monotherapies have suggested that efficacy was higher in patients whose tumors expressed PD-L1 at levels ≥5%, compared with those whose tumors showed lower expression (54). Where the patients were PD-L1-negative however, another study demonstrated that PFS was longer in the combination group (11.2 months) than in the nivolumab alone group (5.3 months) (53). Overall studies to-date support these combination therapies, which appear to benefit patients with low PD-L1 tumor expression.

Studies have also successfully treated melanoma with combined checkpoint blockade regimens where the two antibodies were administered sequentially. A phase III study of two groups of patients with unresectable stage III or IV melanoma investigated patients treated with nivolumab then ipilimumab (*n* = 68), or vice versa (*n* = 70); with nivolumab used as maintenance therapy for both groups until toxicity or disease progression (55). Toxicity was comparable between the groups, however, the nivolumab/ipilimumab exhibited a greater 12-month survival (76%) compared with its counterpart cohort of patients who received the treatments in reverse.

Reducing the dose of ipilimumab in combination with PD-1 directed therapy has resulted in lower combined therapy toxicity rates. Nivolumab with low-dose ipilimumab has been approved in some jurisdictions for the treatment of renal cell carcinoma where toxicity rates have reportedly been reduced compared with combined therapies with higher doses of ipilimumab (56). Research has suggested pembrolizumab as an alternative to nivolumab in a combined regimen with ipilimumab (57). A phase I study, investigating the safety of combining standard-dose (2 mg/kg) pembrolizumab with low-dose (1 mg/kg reduced from 3 mg/kg) ipilimumab, was not powered to examine efficacy (although it suggested comparable level of efficacy to a nivolumab/ipilimumab combination) (57). However, the study demonstrated a more manageable toxicity profile with the pembrolizumab preparation (57).

In summary, the first phase I trials demonstrated higher efficacy than previous monotherapy regimens in small patient cohorts, with a concurrent increase in drug-related toxicity (58). Phase III trials have confirmed these findings showing improved outcomes for advanced-stage melanoma patients when treated with both anti-CTLA-4 and anti-PD-1 therapeutics, which showed benefits for patients with PD-L1 negative tumors (53). Significant toxicity associated with dual therapy limits its usage in patients with comorbidities. However, improved clinical outcomes led to the FDA approval of the combination of nivolumab and ipilimumab for the treatment of unresectable stage III/IV PD-L1 negative melanoma (Table 1). In 2018 the EMA adopted a positive opinion recommending that nivolumab in combination with ipilimumab is indicated for the treatment of unresectable or metastatic melanoma in adults.

## Other Potential Immune Checkpoint Therapy Targets and Combinations With Established Checkpoint Inhibitors

As research into therapies utilizing CTLA-4 and PD-1/PD-L1 blockade to treat melanoma has advanced, further targets have been sought out in an effort to overcome issues such as incomplete tumor regression or relapse following treatment. A concerted effort is underway focusing on inhibitory molecules whose mechanisms may operate within the TME and could have complementary functions to those of approved immunotherapies. Studies in the circulation and tumors of patients with melanoma reveal that the TME promotes T cell, exhaustion demonstrated by upregulation of markers of immunosenescence, thereby allowing T cell impairment and immune escape (59). These markers represent targets for immunotherapy to counteract the immune escape of cancer cells. Targeted treatments combining anti-CTLA-4 or anti-PD-1/PD-L1 alongside blockade of novel checkpoints have the potential to produce comparable effects, perhaps with fewer adverse drug reactions than those of the dual anti-CTLA-4/anti-PD-1 regimens.

### LAG-3/PD-L1 Blockade

The lymphocyte-activation gene 3 (LAG-3) is a co-inhibitory receptor known primarily to be expressed on exhausted TILs which have less potent effector functions (60, 61). LAG-3 may downregulate T cell responses via interaction with major histocompatibility complex class-II (MHC-II) on DCs (61) (Figure 1). Preclinical studies have shown that, as a result of persistent melanoma antigen expression, LAG-3 expression on TILs is increased, thereby inhibiting T cell action and reducing IFN-γ production within the TME under the influence of PD-1 co-stimulation (61). It has been hypothesized that LAG-3 blockade might produce milder side effects than those observed with checkpoint inhibitors currently in clinical use. Autoimmunity developed by *Lag3*^−/−^*Pdcd1*^−/−^ mice (deficient in LAG-3 and PD-1) was slower: approximately 10 weeks compared with 3–4 weeks in CTLA-4 deficient mice, and less penetrant (80% vs. 100%) than the phenotype observed in *Ctla4*^−/−^ mice (60). Indeed, in one phase I trial of LAG3/PD-1 targeting combined therapy, similar safety profiles to nivolumab monotherapy were reported (62). Moreover, *in vivo* studies in murine cancer models have shown that when expressed at high levels, concomitant LAG-3/PD-1 expression is mostly restricted to infiltrating TILs (60). This may signify that a combination immunotherapy targeting these two molecules may encourage tumor-specific responses, avoiding non-specific or self-antigen specific immune responses, perhaps rendering such treatment less toxic than CTLA-4 blockade. Indeed, preclinical evidence that LAG-3 is synergistically efficacious in combination with anti-CTLA or anti-PD-1 therapies is driving clinical development (46).

In addition, PD-L1 overexpression in melanoma tumors has been associated with increased LAG-3 expression. This may point to potential synergistic treatment effects. Pre-clinical studies demonstrated that combination blockade of PD-1 and LAG-3 can induce immune activation and associated tumor rejection in fibrosarcoma and colorectal cancer models in mice (60). LAG-3 has been shown to be expressed on a subset of alternatively-activated human plasmacytoid DCs (pDC). These cells may be enriched in human melanoma tumor sites and produce anti-inflammatory cytokines in response to interactions with MHC class II (61, 63). This may indicate that LAG-3 blockade may promote innate immunity host defense mechanisms. Additionally, melanoma resistance to FAS-mediated apoptosis has been proposed as a mechanism of immune escape mediated by tumor cells expressing MHC class II through engagement with LAG-3 (CD223) expressed on TILs (64). Murine studies have indicated that combined use of anti-LAG-3 and anti-PD-L1 in melanoma treatment overcame the requirement for tumor specific T-reg depletion (65). Because LAG-3 engages with DCs, it is possible that LAG-3 blockade can promote innate immunity, a first host defense mechanism, hence stopping tumor growth at an early stage.

The human IgG4 monoclonal LAG-3 antibody relatlimab is in late phase clinical trials in combination with nivolumab versus nivolumab monotherapy for first line advanced melanoma treatment (Table 3). This regimen holds promise of both efficacy and de-escalation of toxicity.

**Table 3:** Selection of current anti-LAG-3 (relatlimab) clinical trials. Information sourced from ClinicalTrials.gov.

**Trial**	**Aim**	**Target molecules**	**Condition**	**Phase**
NCT02658981	To evaluate the safety and most effective dose of relatlimab or urelumab (CD137) alone and in combination with nivolumab	Relatlimab: anti-LAG-3 mAb Urelumab: anti-CD137 mAb	Recurrent glioblastoma	I
NCT03335540	Evaluate the treatment of solid tumors with various immunotherapy combinations (nivolumab, relatlimab, cabiralizumab, ipilimumab, anti-GITR, IDO1 inhibitor, lirilumab and radiation therapy	Nivolumab: anti-PD-1 mAb Relatimab: anti-LAG-3 mAb Cabiralizumab: anti-CTF1R mAb Ipilimumab: anti-CTLA mAb Lirilumab: anti-KIR mAb Anti-GITR IDO1 inhibitor	Broad biomarker assessment	I
NCT01968109	To assess the safety, tolerability and efficacy of relatlimab alone and in combination with nivolumab in patients with unresectable/metastatic cancer	Nivolumab: anti-PD-1 mAb Relatlimab: anti-LAG-3 mAb	Not previously treated with immunotherapy: • NSCLC • Gastric cancer • Hepatocellular carcinoma • Renal cell carcinoma • Bladder cancer • Squamous cell carcinoma of the head and neck • Melanoma Previously treated with immunotherapy • NSCLC • Melanoma	I/II
NCT02488759	To investigate the safety and effectiveness of nivolumab, and nivolumab combination therapy (relatlimab, ipilimumab and daratumumab)	Relatlimab: anti-LAG-3 mAb Ipilimumab: anti-CTLA mAb Daratumumab: anti-CD38 mAb	Virus associated cancers: • Anal canal cancer • Cervical cancer • Epstein Barr Virus (EBV) positive gastric cancer • HPV positive and negative squamous cell cancer of the head and neck (SCCHN) • Merkel Cell Cancer • Nasopharyngeal cancer (NPC) • Penile cancer • Vaginal and vulvar cancer	I/II
NCT02061761	To evaluate the safety, tolerability and maximum tolerated dose of relatlimab administered alone or in combination with nivolumab to subjects with relapsed hematologic malignancies	Nivolumab: anti-PD-1 mAb Relatimab: anti-LAG-3 mAb	• Relapsed or refractory Hodgkin lymphoma (HL), • Relapsed or refractory Diffuse Large B Cell lymphoma (DLBCL)	I/II
NCT03459222	To investigate safety and anti-tumor activity of relatlimab combination therapy in metastatic/unresectable solid cancers	Relatimab: anti-LAG-3 mAb	Incurable metastatic/unresectable solid tumor excluding CNS metastases	I/II
NCT02750514	To evaluate the efficacy of nivolumab in combination with other agents (dasatinib, relatlimab, IDO1 inhibitor)	Nivolumab: anti-PD-1 mAb Relatimab: anti-LAG-3 mAb Dasatinib: tyrosine kinase inhibitor IDO1 inhibitor	NSCLC	II
NCT02996110	To compare the efficacy and safety of nivolumab combination therapies (relatlimab/IDO1 inhibitor) with nivolumab and ipilimumab	Nivolumab: anti-PD-1 mAb Relatimab: anti-LAG-3 mAb Ipilimumab: anti-CTLA mAb IDO inhibitor	Renal cell carcinoma	II
NCT02935634	To compare the efficacy and safety of nivolumab combination therapies (relatlimab/IDO1 inhibitor) with nivolumab and ipilimumab	Nivolumab: anti-PD-1 mAb Relatimab: anti-LAG-3 mAb Ipilimumab: anti-CTLA mAb IDO inhibitor	Gastric cancer	II
NCT03470922	To compare the efficacy of nivolumab in combination with relatlimab and nivolumab alone	Nivolumab: anti-PD-1 mAb Relatimab: anti-LAG-3 mAb	Unresectable/metastatic melanoma	II/III

### TIM-3/PD-1 Blockade

T cell immunoglobulin and mucin 3 (TIM-3), a co-inhibitory receptor expressed on T cells, has both inhibitory and activating properties (Figure 1). It has been shown to induce T cell apoptosis, anergy and exhaustion via interaction with galectin-9 on immune cells (66). TIM- 3 expressed on TILs has also been found to bind to galectin-9 expressed on tumor cells *in vitro*, promoting immune escape (66). In addition, TIM-3 interactions with the high mobility group box 1 (HMGB1) protein, which is involved in the recruitment of nucleic acids into endosomes to be sensed by the innate immunity, impairs this mechanism promoting tumor escape (67). Since TIM-3 has been established as an exhaustion marker in cancer, it is an attractive immunotherapy target (68). Compared with single agent PD-1 blockade in murine cancer models, it has been shown that combined TIM-3/PD-1 blockade led to superior tumor regression (68). *In vivo* and *ex vivo* research into the properties of TIM-3 has shown that a melanoma peptide vaccine induced CD8+ T cells to upregulate PD-1 and to an extent TIM-3 in immunized patients. Simultaneous TIM-3 and PD-1 blockade enhanced the proliferation of CD8+ T cells (69). Dual anti-TIM-3 (MBG453) plus anti-PD-1 (PDR001) blockade is currently being analyzed in phase I/II trials (NCT02817633, NCT02608268, NCT03099109, NCT03066648).

### B7-H3/CTLA-4 Blockade

B7-H3 (CD276) is a receptor of the CD28 (a co-stimulatory molecule) and B7 (a co-inhibitory molecule) family molecules found on APCs (Figure 1). B7-H3 has been found to be over-expressed in melanoma, favoring tumor growth and conferring anti-apoptotic processes (70). When targeted by an Fc-optimized anti-B7-H3 (MGA271) humanized IgG1 antibody, potent antibody-dependent cellular cytotoxicity (ADCC) against melanoma *in vitro* and *in vivo* was observed (71). Studies have suggested than B7-H3 blockade could suppress its co-stimulatory properties. Anti-sense oligonucleotides shown to inhibit B7-H3 expression on DCs resulted in inhibition of IFN-γ production by DC-activated T cells and the proliferation of T cells *in vitro* (72). Subsequently, ipilimumab plus enoblituzumab, a first in class mAb targeting B7-H3, have been the subject of a phase I trial (NCT02381314), and also enoblituzumab in combination with pembrolizumab is being tested in refractory cancers (NCT02475213) (73).

### VISTA/PD-1 Blockade

V-domain Ig suppressor of T cell activation (VISTA) is a PD-L1 homolog and co-inhibitory receptor of the B7 family predominantly expressed on various hematopoietic cells (myeloid derived suppressor cells (MDSCs), tumor associated macrophages (TAMS) and DCs) (74) and on leukocytes such as naïve T cells (Figure 1). Particularly high levels of VISTA were found on tumor infiltrating myeloid cells in murine models (75). VISTA may participate in the suppression of T-eff responses and T-reg induction via interaction with its putative ligand VSIG-3 (75, 76). VSIG-3 is thought to inhibit T cell function and, in the presence of TCR signaling, it may impair T cell proliferation via the VSIG-3/VISTA pathway (77). As well as expression on T cells, it has also been noted to be upregulated in tumors such as colorectal cancer or hepatocellular carcinoma (77). A murine study using B16-OVA melanoma cell lines demonstrated that VISTA blockade with a monoclonal antibody (13F3) enhanced T-eff response within the TME (78). Therefore, blockade of VISTA could enhance innate immunity since it is expressed on myeloid cells thereby promoting early melanoma eradication. VISTA is also expressed on naïve T cells, and inhibition of VISTA could promote early T cell reaction in response to tumor cells (79). VISTA can control T cell activation through nonredundant functions distinct from the PD-1/PD-L1 pathway in controlling T cell activation and antibodies targeting both checkpoints have shown efficacy in preclinical models (80). Therefore, concurrent targeting VISTA and PD-1 pathways has been proposed as a therapeutic approach. The small molecule antagonist CA-170 electively targets PD-L1/2 and VISTA and is presently tested in a phase I trial (NCT02812875) in advanced solid tumors and lymphomas.

### A2AR/PD-L1 Blockade

The adenosine-adenosine A2A receptor (A2AR) pathway is of interest as a target for immunotherapy, since the extracellular adenosine, often found in TME due to hypoxia, can inhibit T cell proliferation and cytotoxicity via the A2AR receptor (CD73) on myeloid cells such as macrophages (81) (Figure 1). CD73 is a checkpoint molecule expressed on T-reg which converts AMP to adenosine in this pathway and this checkpoint is also expressed on melanoma cells. Studies of patient samples *ex vivo* have shown that via adenosine interaction, tumor cells are able to inhibit immune responses and to simultaneously enhance neovascularization and cancer cell growth via vascular endothelial growth factor (VEGF) and IL-6 expression (82, 83). Synergistic effects of combined CTLA-4 and CD73 as well as combinations of PD-1 and CD73 blockade immunotherapies in breast cancer and colon carcinoma pre-clinical models (84, 85) have been observed. Furthermore, murine studies have demonstrated suppressed melanoma growth and enhanced lymphocytic infiltration in the TME in A2AR-deficient mice. Early phase trials investigating the merits of a combined anti-CD73/anti-PD-L1/anti-PD1 therapies in patients with solid tumors are underway, to ascertain whether or not targeting multiple sites in the pathway can show enhanced anti-tumor effects (e.g. NCT02655822, NCT02503774) (Table 4).

**Table 4:** Selection of current A2AR/CD73 inhibitor clinical trials. Information sourced from ClinicalTrials.gov.

**Trial**	**Aim**	**Target molecules**	**Condition**	**Phase**
NCT03454451	To evaluate the safety, tolerability, and anti-tumor activity of CPI-006 as a single agent, in combination with A2AR inhibitor CPI-444 and in combination with pembrolizumab	CPI-006: CD23 and adenosine inhibitor CPI-444: A2AR inhibitor Pembrolizumab: humanized anti-PD-1 mAb	• Non-small cell lung Cancer (NSCLC) • Renal cell cancer • Colorectal cancer • Triple negative breast cancer • Cervical cancer • Ovarian cancer • Pancreatic cancer • Endometrial cancer • Sarcoma • Squamous cell Carcinoma of the head and neck • Bladder cancer • Metastatic castration Resistant prostate cancer	I
NCT02503774	To evaluate the safety, tolerability, pharmacokinetics, immunogenicity, and antitumor activity of MEDI9447 (oleclumab) alone and in combination with MEDI4736 (Durvalumab) in adult subjects with select advanced solid tumors	Oleclumab: human anti-CD73 mAb Durvalumab: human anti-PD-1 mAb	• Advanced solid malignancies • NSCLC	I
NCT02655822	To study the safety, tolerability, and anti-tumor activity of A2Ar inhibitor CPI-444 alone and in combination with atezolizumab	CPI-444: A2AR inhibitor Atezolizumab: humanized anti-PD-1 mAb	• Non-small cell lung cancer • Malignant melanoma • Renal cell cancer • Triple negative breast cancer • Colorectal cancer • Bladder cancer • Metastatic • Castration resistant Prostate cancer	I
NCT02740985	To determine the maximum tolerated dose of A2AR receptor antagonist AZD4635 in combination with durvalumab	Durvalumab: human anti-PD-1 mAb AZD4635: A2AR receptor antagonist	• Advanced solid malignancies • NSCLC • Metastatic castrate-resistant prostate carcinoma • Colorectal carcinoma	I
NCT03549000	To assess the safety, tolerability, and anti-tumor activity of anti-CD73 NZV930 alone and when combined with anti-PD1 and/or A2AR inhibitor NIR178	NZV930: anti-CD73 NIR178: A2AR inhibitor PDR001: experimental anti-PD-1	• NSCLC • Triple negative breast cancer • Pancreatic ductal adenocarcinoma • Colorectal cancer microsatellite stable • Ovarian cancer • Renal cell carcinoma	I
NCT02403193	To determine the safety, tolerability, feasibility and efficacy of A2AR inhibitor PBF-509 alone and in combination with anti-PD1	PBF-509: A2AR inhibitor PDR001: experimental anti-PD-1	NSCLC	I/II
NCT03381274	To evaluate the safety, tolerability and antitumor activity of novel combination therapies (oleclumab, A2AR inhibitor AZD4635 and osimertinib)	Oleclumab: human anti-CD73 mAb AZD4635: A2AR inhibitor Osimertinib: tyrosine kinase inhibitor	NSCLC	I/II
NCT03207867	To evaluate the efficacy and safety of A2AR antagonist (NIR178) in combination with anti-PD1	NIR178: A2AR inhibitor PDR001: experimental anti-PD-1	• NSCLC • Renal cell cancer • Pancreatic cancer • Urothelial cancer • Head and neck cancer • Diffused large B cell • Lymphoma • Microsatellite stable colon cancer • Triple negative breast cancer • Melanoma	II

### IDO/CTLA-4, PD-1 and PD-L1 Blockade

Indoleamine 2'3' dioxygenase (IDO, IDO1 and IDO2) is a catabolic enzyme produced by macrophages and T-regs to convert tryptophan, which is needed for T cell effector function, to kynurines (86) (Figure 1). Immune tolerance is promoted by IDO-mediated tryptophan deficiency, with naïve T cells observed to differentiate into T-reg (87). This protein can also be expressed by tumors alongside prostaglandin E2, thereby aiding in immune escape of these cancers via NK cell inhibition (88) and T-reg recruitment resulting in IL-10 mediated induction of myeloid-derived suppressor cells (MDSC) (89). *Ex vivo*-derived and tumor-associated MDSC have been shown to express PD-L1 and MHC-II, and correlated with expression of their receptors, PD-1 and LAG- 3, on T cells, known to be associated with immunosuppression of T cell functions. PD-L1-expressing MDSCs could trigger immunosuppressive effects via IDO (90). Importantly, a pre-clinical study in a melanoma model in mice demonstrated IDO overexpression post-treatment with anti-CTLA-4 and anti-PD-1 (14). This conferred resistance and tumor growth, a property found to be reversible by combination treatment with anti-CTLA-4 and IDO inhibitors (14). Studies using the murine B16.SIY melanoma mouse model have shown that combinations of CTLA-4 or PD-1/PD-L1 with IDO blockade restored both IL-2 production and CD8+ T cell proliferation within the TME (48), pointing to the potential merits of a combinational targeting approach.

IDO and galectin-3 expression are known to promote T-reg upregulation, while suppressing T-eff production (91, 92). Blockade of these two molecules reversed these effects (91), and although further investigations into combined anti-PD-1/IDO (epacadostat) inhibitors underway in a phase I and II trial (NCT02318277) were promising, a phase III trial studying an epacodostat-pembrolizumab combination was abandoned due to a lack of significant improvement in both primary and secondary endpoints—PFS and overall survival (OS) (93). Epacodostat has had to be discontinued in several trials assessing its efficacy as a monotherapy including KEYNOTE-006, KEYNOTE-010, KEYNOTE-087 and KEYNOTE-052, due to adverse reactions (94). These disappointing results may influence the design of future studies aiming to assess IDO as a potential immunotherapy target. However, different approaches to direct IDO inhibition, including an IDO peptide vaccine, have been promising and have shown significant delay in tumor growth and prolonged survival in a B16 murine model (95). Early phase trials of combinations such as a PD-L1/IDO peptide vaccine with nivolumab (NCT03047928) in advanced melanoma are underway.

### PKC-η/PD-1 Blockade

A protein which may synergize with CTLA-4 to mediate immune tolerance is PKC-η, an intrinsic downstream signaling checkpoint molecule (96) (Figure 1). PKC-η induces anti-inflammatory cytokine transcription by activating NFkB downstream of the cascade (97). Downregulation of PKC-η in murine models reduced the tumor-suppressive activity of T-reg but did not enhance autoimmune colitis (96). It is possible that inhibition of PKC-η could potentially produce effective T-reg suppression and with fewer adverse drug reactions if used in combination with anti-CTLA-4 blockade. Pre-clinical studies on Rag1 mice have shown that a PKC-η deficiency reduced tumor growth (98) and there are currently studies investigating the efficacy of midostaurin, a tyrosine kinase inhibitor, for the treatment of leukemia as well as studies on the pan-PKC inhibitor AEB071 sotrastaurin which is being tested in uveal melanoma (NCT02273219, NCT02601378, and NCT01430416).

## Antibody Engineering and Novel Combinations to Improve Checkpoint Blockade

**Figure 2:**
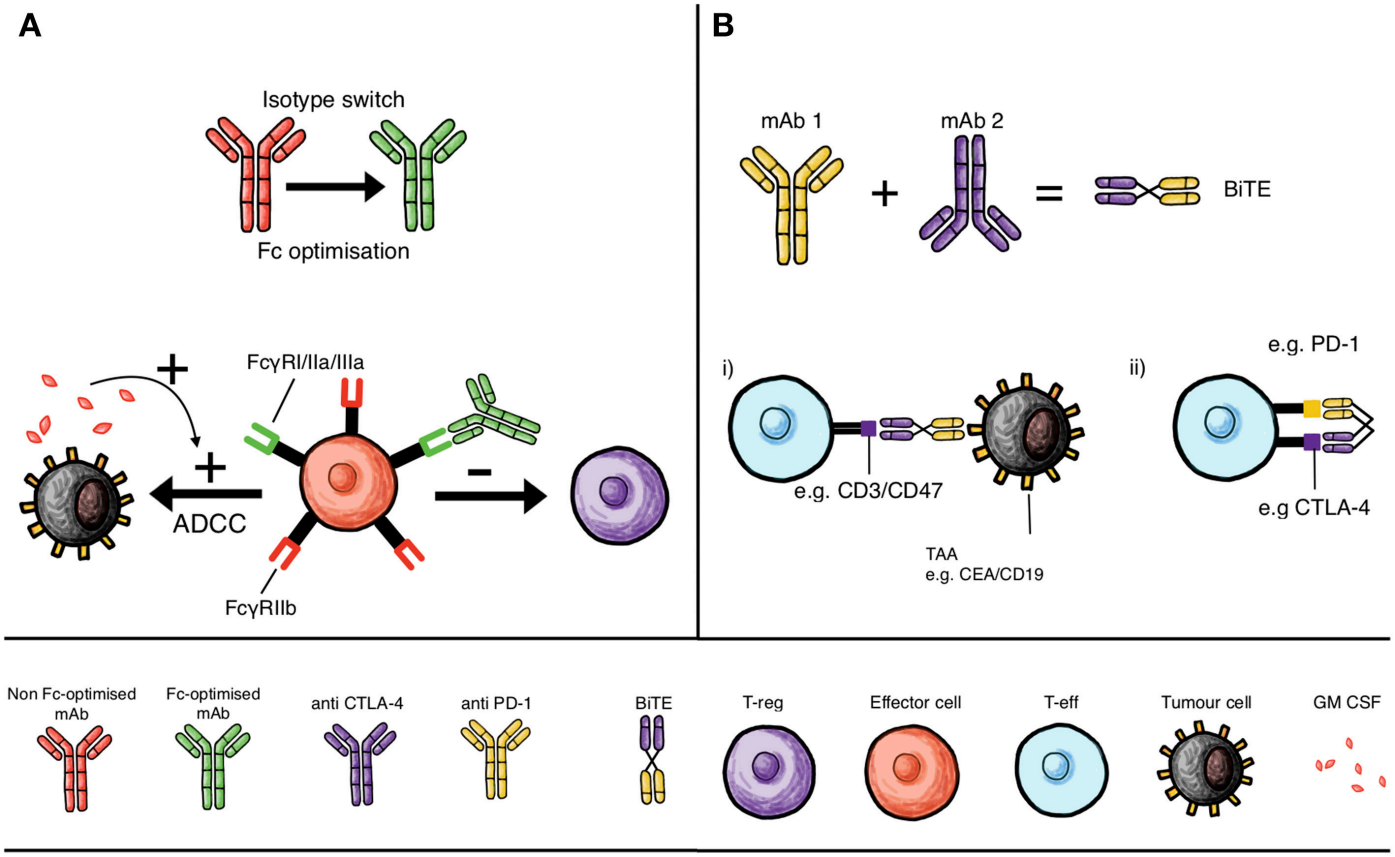
Monoclonal antibody mechanisms of action and interactions with immune cells that can influence checkpoint inhibition: isotype switching, Fc domain optimization and BiTEs. **(A)** Isotype switching and Fc optimization of mAbs to increase binding to activatory Fc receptors on effector cells, such as monocytes, macrophages and NK cells, to enhance T-reg depletion by ADCC, and in the presence of GM-CSF, to enhance ADCC of tumor cells. **(B)** Development of bispecific T cell engaging antibody structures (BiTE) that can either simultaneously engage tumor-associated antigen (TAA, e.g., CEA or CD19) and T cell specific molecules (e.g., CD3 or CD47) to promote cytolysis of tumor cells (**B**,i) or simultaneously inhibit two T cell checkpoint molecules; such as CTLA-4 and PD-1, to circumvent mechanisms of resistance (**B**,ii).

Combining checkpoint blockade antibodies may be an effective strategy for treating melanoma (Figure 1). However, there remain numerous avenues available for the refinement of existing therapies. With advances in protein engineering, antibodies can be manipulated to introduce novel functionality (99). For combined checkpoint blockade therapies, there is evidence that such engineering could further improve clinical efficacy. Anti-CTLA-4 antibodies have been observed in mouse models of cancer to deplete T-reg cells by ADCC, a function that relies on specific antibody isotypes engaging with FcγR on effector cells (monocytes, macrophages, NK cells) that are cytotoxic to T-regs within the TME (20, 100) (Figure 2A).

Until recently, there has been an assumption that Fc receptors would not contribute to the anti-tumor activity of antibodies recognizing checkpoint molecules (101). FcγRs expressed mainly on hematopoietic cells such as NK cells, DCs and macrophages (102) can activate or inhibit; in particular, FcγRI, FcγRIIa, and FcγRIIIa are activating receptors while FcγRIIb is inhibitory (101) (Figure 2A). Importantly, FcγRs are co-expressed on the same cells such as monocytes and macrophages, allowing thresholds for activation/inhibition to be fine-tuned (101). Ipilimumab, for instance, exhibits T-reg depletion properties specifically in the presence of FcγR-expressing monocytes and natural killer (NK) cells, consistent with clinical studies that have demonstrated correlations between specific checkpoint inhibitor antibody subtypes of activating hFcγRs and clinical responses (100) (Figure 2A). An example of this includes variants in the gene *FCGR (FCGR2A* and *FCGR3A*) which bind more avidly to human IgG1 and IgG2 subtypes, thereby increasing ADCC-mediated cell death and thus have been associated with improved outcomes (100). Whether these single nucleotide polymorphisms (SNPs) can affect the response of other immunomodulatory monoclonal antibodies requires further investigation.

*In vitro* studies of anti-CTLA-4 monoclonal antibodies with the same IgG1 and IgG2 Fc variants such as ipilimumab and tremelimumab exhibited superior tumor killing with antibodies able to maximize T-reg depletion by ADCC (20, 100) (Figure 2A). Additionally, preclinical studies have shown that targeting CTLA-4 on T-reg cells conferred minimal tumor protection in comparison to when antibody bound to CTLA-4 on both T-eff and T-reg compartments *in vivo* (101). Preclinical studies demonstrated a requirement for enriching the TME with effector cells such as myeloid cells expressing high levels of FcγRs (103). One phase II trial showed that granulocyte–macrophage colony-stimulating factor (GM-CSF) plus ipilimumab improved overall survival in patients with metastatic melanoma compared with ipilimumab alone, possibly as a result of a macrophage enriched TME. These findings suggest that macrophages, may be key effector cells in mediating ADCC (101, 103) (Figure 2A).

One molecule of note is CD25, highly expressed on T-reg cells in the TME in mouse models and in human tumors, but with minimal expression in the effector cell compartment (104). Anti-CD25 antibody-induced ADCC can be enhanced by optimizing the antibody isotype to engage activating FcγRs in mouse models of cancer (104). Experiments on mouse models have also shown that anti-CD25 therapies can work concurrently with other immunomodulatory drugs such as anti-PD-1 monoclonal antibodies in the TME. Fc-optimized anti-CD25 drug combinations may prove promising through improving the drug therapeutic window (104). However, it is important to note that CD25 is also expressed at lower levels on activated memory T cell populations (105) potentially highlighting a risk for unwelcome T-eff depletion with anti-CD25 treatment.

Bispecific T cell engaging antibodies (BiTE) constitute an extensive class of agents able to recognize a range of tumor antigens expressed on cancer cells on one arm, while simultaneously engaging a T cell-specific molecule such as CD3 through the other arm (Figure 2B). BiTEs are able to link the two, promoting cytolysis of tumor cells. A bispecific T cell engaging (BiTE) antibody which recognizes the tumor antigen carcinoembryonic antigen (CEA) with one arm and CD3 with the other has been suggested for improving checkpoint blockade therapies (106) (Figure 2B). Following BiTE-mediated cytolysis, upregulation of PD-1 impaired T cell functions (107). If the BiTEs are delivered in conjunction with PD-1 checkpoint blockers, these T cell modulating effects may be reversed (107). It is therefore possible that checkpoint blockers could synergize with such cancer immunotherapies when used in combination.

Bispecific antibodies have also been generated which target checkpoint molecules directly. CD47 is an immune-regulatory molecule expressed on the surface of many cells and all human cancers. It can prevent phagocytosis of cancer cells by macrophages and dendritic cells by binding to SIRPα on their surface (108). However, the ubiquity of CD47 makes it difficult to specifically target with antibodies. Considering this, a bispecific antibody recognizing both CD47 and a cancer-specific antigen, in this case CD19 for B cell lymphoma, has been generated. *In vitro*, this antibody could promote effective phagocytosis of the cancerous cells (109). It is possible to imagine that this targeted checkpoint blockade approach may be exploited for use melanoma, provided an appropriate targeting antigen can be identified. Furthermore, a BiTE designed to simultaneously target two immune checkpoints on T-eff cells, such as CTLA-4 and PD-1, may improve tumor killing and increase efficacy since dual therapies of anti-PD-1 and anti-CTLA-4 have proven effective (Figure 2B). This could circumvent mechanisms of resistance and eschew the need for two mAbs to be administered concurrently.

**Figure 3:**
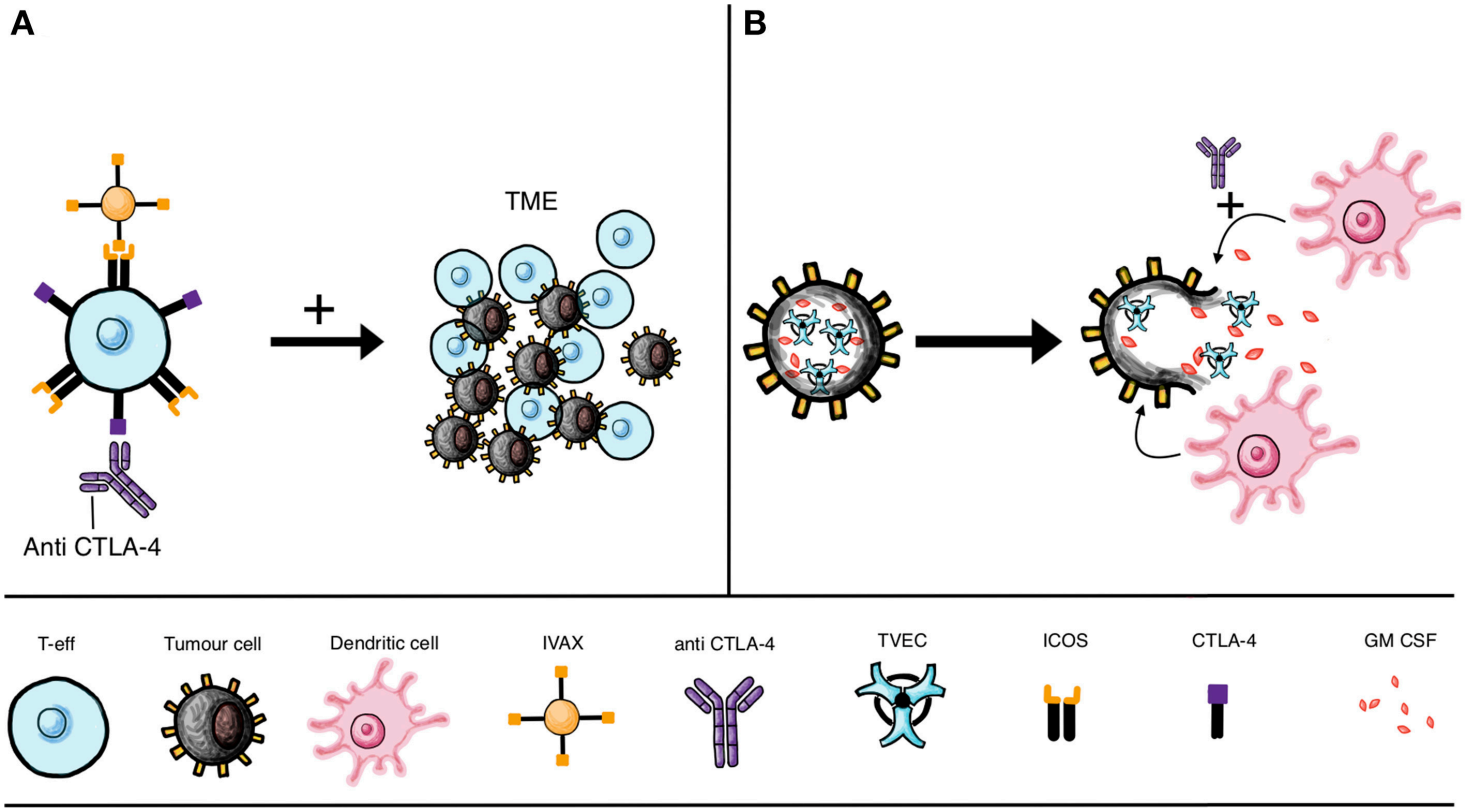
IVAX vaccine and oncolytic viruses combined with checkpoint blockade. **(A)** Combinatorial therapy may include CTLA-4 blockade with anti-CTLA-4 mAb (leading to T cell activation and subsequent ICOS upregulation) together with the IVAX vaccine [which engages ICOS to increase T-eff migration to the tumor microenvironment (TME)]. **(B)** Approaches beyond antibody engineering include attenuated oncolytic virus, such as T-VEC unable to replicate in healthy cells, instead preferentially invading cancer cells. The replicating virus lyses the tumor cells and is armed, for instance with GM-CSF, which when released can recruit DCs to prime T cells to identify and destroy tumor cells. Efficacy of oncolytic virus may be improved by combination with checkpoint inhibitors, such as anti-CTLA-4 treatment to boost T cell activation.

T cell activation upregulates ICOS expression and targeting the ICOS/ICOSL pathway improved the potency of anti-CTLA-4 therapy in preclinical models (110, 111). The cellular vaccine IVAX (irradiated ICOSL-positive tumor cells) has been shown to function synergistically in the context of CTLA-4 blockade. The combination has been shown to lead to effector cell migration to and survival in the TME, and consequent enhanced tumor elimination in murine models (110) (Figure 3A).

### Beyond Antibody Engineering: Oncolytic Viruses as Immunotherapies

Oncolytic viruses (OVs) are being developed as cancer immunotherapies, with the modified herpes simplex virus type 1 talimogene laherparepvec (T-VEC, or OncoVEX^GM−CSF^) being approved by the FDA for malignant melanoma (112), and the ClinicalTrials.gov database currently listing 22 trials studying oncolytic herpes simplex virus following the success of T-VEC for metastatic melanoma (113) (Figure 3B). Many tumor cells cannot adequately protect themselves against viral infection, making the development of oncolytic agents an attractive therapy option that may selectively infect tumor cells (113–115). Among the properties of OVs is their ability to improve the immune system response to tumors, and in combination with checkpoint inhibitors they also enhance checkpoint blockade (116–118). By lysing tumor cells, OV treatment can lead to the release of tumor-associated antigens by the dying cells and efficient cross-presentation of antigens to DCs, thus enhancing anti-tumor responses (113, 116) (Figure 3B).

In clinical practice, OVs are made cancer specific by attenuating the virus so that preferential infection of tumor cells occurs (119). In addition to this, cytokine expression of OVs may increase the anti-cancer properties of these therapies avoiding systemic toxicity due to the preferential action of OVs on tumor cells (119). IL-12 in particular is an important cancer immune response modulator and oncolytic herpes viruses (oHSVs) expressing IL-12 have shown efficacy in preclinical studies on glioblastoma multiforme (120). ‘Arming’ these OVs with an expression of a particular cytokine may be key for achieving anti-tumor efficacy. The clinically-licensed T-VEC for instance is armed with GM-CSF, an APC activator (121). A murine study also found that an IL-12 armed oHSVs delayed tumor proliferation and reduced tumor growth more effectively than its unarmed counterpart (119) (Figure 3B).

Although viral-mediated tumor lysis can be enhanced by tropism targeting and viral arming with immune stimulatory cytokines, clinical efficacy has not been consistent across all patient groups (122). For instance, the cytidine deaminase Apolipoprotein B Editing Complex 3 (APOBEC3), normally involved in the host response to retrovirus infection, is also reported to be associated with virus-resistant tumors. Expression of APOBEC3 been shown to be upregulated in B16 murine melanoma cells infected with an oncolytic vesicular stomatitis virus (VSV) (122). Tumor cell resistance to VSV both *in vivo* and *in vitro* was demonstrated where APOBEC3 was upregulated and knockdown of this enzyme reduced VSV escape from immune clearance (122). This highlights the need for further research to clarify methods for mitigating and preventing tumor resistance to immunotherapies and other novel therapies that often limit efficacy.

Studies have suggested the potential merit of combining OVs with checkpoint inhibitors. In a Phase II study, CD8-expressing T cells, immune activation markers and PD-L1 expression were increased with intralesional administration of coxsackievirus A21 (CVA21), which has a propensity for tumor cells expressing ICAM-1 (123). Phase Ib trials have shown that this combination has minimal additional toxicity or adverse drug reactions, with one study recording an ORR of 73% and a disease control rate (DCR) of 91% of patients with advanced melanoma (124, 125). In the case of T-VEC, long term protection and significant effects on untreated tumors was seen only when the OV was combined with anti-CTLA-4 (121). Finally, several studies are exploring novel OV combinations. One is an oncolytic vaccinia virus armed with a superagonist IL-15 (IL-15-IL-15Rα fusion) given alone or in combination with anti-PD-1: combined treatment demonstrated T cell-driven anti-tumor efficacy in *in vivo* mouse models of cancer (126). Furthermore, TNFα armed viruses have seen success in inducing tumor regression and vascular collapse in solid masses when administered in tumor-bearing mice alongside therapies that inhibit anti-apoptosis proteins *in vitro* (127).

The administration of OVs has often been limited to intratumoral delivery in patients; T-VEC is currently only licensed as a locally-administered treatment (128). This is due to concerns regarding neutralizing antibodies (NAbs) which are often present in human populations in response to common viruses such as HSV and reovirus and may impair the efficacy of systemic delivery of OVs (128). This may be a limitation since systemic delivery of OV therapy would in theory be more effective for targeting metastatic lesions. However, one report (128) found that by loading neutralized reovirus with antibodies in immune complexes onto human monocytes, resulted in the transfer of the virus to melanoma cells, cancer cell lysis and in restriction of cancer growth in a murine model of melanoma (128). This indicates that monocytes and antibodies recognizing viral proteins may be important components in maintaining the potency of OV therapy and may lead to new strategies for OV treatment.

In summary, oncolytic viruses may provide an alternative avenue for cancer immunotherapy due to their properties in targeting cancer cells with some specificity and due to some promising results in combination with immune checkpoint blockade. It appears that these therapies are most efficient when armed with specific cytokine expression and attenuation of the virus is important for both reducing toxicity and allowing preferential infection of cancer cells.

## Challenges and Future Directions

Anti-CTLA-1 and anti-PD-1 therapies have been the most extensively researched due to documented benefits for melanoma treatment as evidenced by higher rates of tumor clearance and, crucially, long term disease eradication in some patients compared with traditional treatments (129). Importantly, further research has emphasized improved response and survival rates when combining anti-CTLA-4 and anti-PD-1 therapy (130), leading to the approval of ipilimumab plus nivolumab in BRAF wild-type melanoma in 2015 and regardless of BRAF tumor subtype in 2016 (131). As more and more immune checkpoints are identified, and their mechanisms of action elucidated, new options for single and combined therapy regimens can be derived (Table 5). Combining different checkpoint blockade agents may produce potent effects, due in part to targeting more than one immune pathways. The benefits of combination therapies are not limited to the use of multiple checkpoint inhibitors but can extend to combining anti-CTLA or anti-PD-1 drugs with other targeted agents such as MEK and BRAF inhibitors. One such example is a Phase III trial combining PD-L1 and MEK inhibition (atezolizumab and cobimetinib) vs. pembrolizumab in advanced BRAF^V600^ wild-type melanoma (NCT03273153). Combinations of checkpoint inhibitors with chemotherapy, radiotherapy and other immunotherapies such as oncolytic viruses and cell-based therapy approaches are also being investigated. Many challenges, including selecting optimal combinations, predicting and managing toxic effects for different combinations and development of clinically-useful biomarkers to help support the use of such treatments are and will continue to be the subject of intense study.

**Table 5:** Examples of combination phase III monoclonal antibody immunotherapy trials in melanoma. Information sourced from ClinicalTrials.gov.

**Indication**	**Trial number**	**Combination therapy**	**Study design**	**Target**	**Status**
**DUAL MONOCLONAL ANTIBODY THERAPIES**					
Previously untreated, unresectable or metastatic melanoma	NCT02905266	Nivolumab + ipilimumab	Arm A: nivolumab and ipilimumab concomitant administration followed by nivolumab monotherapy Arm B: nivolumab and ipilimumab sequential administration followed by nivolumab monotherapy	Anti-PD-1 + anti-CTLA-4	Active, not recruiting
Complete resection of stage IIIB/C/D or stage IV melanoma	NCT03068455 (CheckMate 915)	Nivolumab + ipilimumab	Arm A: nivolumab + ipilimumab Arm B: nivolumab	Anti-PD-1 + anti-CTLA-4	Active, not recruiting
Previously untreated, unresectable or metastatic melanoma	NCT02714218	Nivolumab + ipilimumab	Arm A: nivolumab 3 mg/kg IV + ipilimumab 1 mg/kg IV Arm B: ipilimumab 3 mg/kg IV + nivolumab 1 mg/kg IV Arm C: nivolumab 6 mg/kg IV + ipilimumab 1 mg/kg	Anti-PD-1 + anti-CTLA-4	Active, not recruiting
First-line for advanced melanoma	NCT02599402 (CheckMate 401)	Nivolumab + ipilimumab	Arm A: nivolumab + ipilimumab Arm B: nivolumab	Anti-PD-1 + anti-CTLA-4	Active, not recruiting
Unresectable or metastatic melanoma	NCT03470922	Nivolumab +relatlimab	Arm A: relatlimab + nivolumab Arm B: nivolumab	Anti-PD-1 + LAG-3 inhibitor	Recruiting
**COMBINED MONOCLONAL ANTIBODY AND TYROSINE KINASE INHIBITORS**					
Stage III-IV BRAF^V600^ melanoma	NCT02224781	Ipilimumab and nivolumab + dabrafenib and trametinib	Arm A: ipilimumab and nivolumab then dabrafenib and trametinib Arm B: dabrafenib and trametinib then ipilimumab and nivolumab	Anti-CTLA-4 and anti-PD-1 + BRAF inhibitor and MEK inhibitor	Recruiting
Previously untreated BRAF^V600^ mutation-positive patients with metastatic or unresectable locally advanced melanoma	NCT02908672	Atezolizumab + cobimetinib + vemurafenib	Arm A: atezolizumab + cobimetinib + vemurafenib + vemurafenib placebo Arm B: atezolizumab placebo + cobimetinib + vemurafenib	Anti-PD-1 + MEK inhibitor + BRAF inhibitor	Active, not recruiting
Previously untreated advanced BRAF^V600^ wild-type melanoma	NCT03273153	Atezolizumab + cobimetinibPembrolizumab	Arm A: atezolizumab + cobimetinib Arm B: pembrolizumab	Anti-PD-L1 + MEK inhibitorsAnti-PD-1	Recruiting
**MONOCLONAL ANTIBODY THERAPIES COMBINED WITH OTHER AGENTS**					
Anti-PD-1 refractory melanoma	NCT03445533 (ILLUMINATE-301)	Ipilimumab + IMO-2125	Arm A: ipilimumab Arm B: ipilimumab + IMO-2125	Anti-CTLA-4 + TLR9 agonist	Recruiting
Unresectable or metastatic melanoma	NCT02752074 (Keynote-252 / ECHO-301)	Pembrolizumab + epacadostat	Arm A: pembrolizumab + epacadostat Arm B pembrolizumab + placebo	Anti-PD-1 + IDO1 inhibitor	Active, not recruiting
Untreated unresectable stage III or IV melanoma	NCT00324155	Dacarbazine + ipilimumab	Arm A: dacarbazine + ipilimumab Arm B: dacarbazine + placebo	Chemotherapy alkylating agent + anti-CTLA-4	Completed
Unresected melanoma	NCT02263508 (KEYNOTE-034)	Pembrolizumab + T-Vec	Arm A: pembrolizumab + talimogene laherparepvec Arm B: pembrolizumab + placebo	Anti-PD-1 + oncolytic herpes virus	Active, not recruiting
Unresectable or metastatic melanoma	NCT03301636 (NLG2107)	Pembrolizumab/nivolumab + indoximod	Arm A: pembrolizumab + indoximiod Arm B: pembrolizumab + placebo Arm C: nivolumab + indoximiod Arm D: nivolumab + placebo	Anti-PD-1 + IDO inhibitor	Recruiting

Toxicities of immunotherapies remains an important challenge. Adverse effects (50, 62), can be managed according to information from resources such as the European Society for Medical Oncology (ESMO) Clinical Practice Guidelines (132, 133). It has been shown that recurrence of immune-related adverse events (irAEs) in patients in whom it was necessary to discontinue treatment as a result of irAEs can occur upon re-challenge with immunotherapies such as anti-PD-1. For instance, a retrospective analysis showed that colitis was less likely to recur in comparison to hepatitis, pancreatitis, pneumonitis and nephritis, and re-challenge with PD-1 blockade has been reported to be tolerated better than other agents (134). Studies have also suggested various biomarkers such as IL-17 or eosinophilia to help predict toxicity in patients, something that could allow early recognition of pathology and thus prompt intervention (133, 135). Investigation into new immunotherapies and combinations may also reveal treatment algorithms with more favorable toxicity profiles than the current regimens.

Improved efficacy has often been associated with increased irAEs, as reported in trials such as Checkmate 067, where the ipilimumab/nivolumab combination had a 10% higher rate of toxicity than either drug administered alone (52). However, immune-related toxic effects may potentially be abrogated in future therapeutic regimens by various approaches. These may include the increasing specificity of targeted immune therapies, to help reduce systemic effects on non-target healthy cells, as well as the implementation of combinatorial therapies with non-overlapping targets. For instance LAG-3 may promote more tumor-specific responses than currently licensed immunotherapies: concomitant LAG-3/PD-1 expression is mostly restricted to infiltrating TILs, expressed at high levels in murine models (60), and mouse studies into other combinations such as a PKC-η/PD-1 blockade therapy has shown a reduction in T-reg immunosuppressive activity without the concomitant increase in autoimmunity (96). Thus, although data from current licensed therapies have suggested a correlation between combinatorial therapies and increased toxicity, this may not necessarily be the case with different checkpoint blockade combinations. In the future, ideally patients would be able to access fully personalized medicine in which treatment such as immunotherapies would be specific to the patient immune response as well as the specific genetic characteristics of their cancers—this would reduce unwanted side effects and maximize the antitumor effect in each individual patient.

The mechanisms behind the lack of clinical responses to single or combination treatments in many patients, how tumors develop resistance to novel immunotherapies and the best criteria to select patients for maximum treatment effect are insufficiently elucidated. A focus on functional evaluations in the context of patient immunity for novel regimens alongside clinical testing may shed some light on the most effective combinations and the best strategies to reduce adverse effects. This may reveal immune or genomic biomarkers linked with better treatment outcomes. For instance, cancer development and therapeutic response may relate to host factors such as the gut microbiome. Approximately 20% of malignancies are linked with microorganism infection and a patient's gastrointestinal microbiota can both positively and negatively influence cancer susceptibility (136). The effects of the microbiome in cancer susceptibility, progression and response to treatment are far reaching insofar as the microbiome is known to guide the immune system's response, host metabolism of medication and endogenously produced chemicals, and can also influence the balance of cell growth and death (136). A recent study of patients with melanoma exploring the role of the microbiome in influencing clinical response to anti-PD-1 therapy found that “favorable” gut microbiota (characterized by higher gut microbe diversity and levels of Ruminococcaceae/Faecalibacterium) mediated higher levels of antigen presentation and T-eff function both in the periphery and within the TME. This promoted better systemic and anti-tumor immune responses compared with patients with “unfavorable” microbiomes (137). Bacteroides has been linked to a poorer anti-tumor response in patients treated with ipilimumab and patient microbiomes enriched with Faecalibacterium and Firmicutes have also been correlated with more efficacious clinical response to ipilimumab (138).

Research exploring predictive markers for checkpoint treatment response has pointed to mutational burden, PD-1 ligand (PD-L1) expression, circulating tumor cells (CTCs) and miRNA signatures. The challenge of identifying biomarkers should also be explored in combination therapies. For melanoma, mutational burden and PD-L1 studied in pretreatment biopsies have been evaluated as predictive markers for guiding therapy. However, these do not always correlate with response (139). A study exploring a noninvasive blood-based monitoring method of tumor burden, aimed at improving prediction of response for melanoma patients undergoing immune checkpoint inhibition therapy, found longitudinal digital measurements of circulating tumor cells (CTCs) to be predictive of clinical outcomes in patients with metastatic melanoma (139). Other research studies have reported falling levels of circulating free DNA (ctDNA) for tumor survival-promoting mutant kinase BRAF, NRAS, and KIT alleles in melanoma patients who are undergoing immunotherapy (140, 141). Alongside their use as biomarkers of response, perhaps an optimal immune and mutant kinase targeted therapy combination could be derived to further improve clinical outcomes. Tumor lymphocyte and NK cell infiltration and IFNγ upregulation, have been proposed as potential predictors of response alongside mutational burden, however these need to be standardized and widely evaluated in clinical practice (142–144). Tumor mutational load and PD-L1 expression are often clinically available to physicians. PD-L1 expression has been reported to have a good negative predictive value in lung cancer; however, the same results have not been shown in melanoma (142). Finally, new technologies such as analyzing T cell clonality are promising but require specialist equipment not yet widely available as clinical tools (142). Reliable predictive markers of long term outcome are also particularly important for combined therapies with their high toxicity profiles. Identifying signatures to guide selection of patients who do not need to be exposed to the increased risk of toxicity currently inherent in combination strategies would greatly aid clinical decision making.

## Conclusion

Combinations of existing and novel immune checkpoint inhibitors and discovering predictive biomarkers promise to further build on the success of immunotherapy. A comprehensive approach will be required to produce the most efficacious combination immunotherapies able to circumvent mechanisms of resistance. Antibody engineering may help capitalize on tumor killing effector mechanisms through knowledge of SNPs and FcR-expressing immune effector cells known to influence patient responses to other immunomodulatory monoclonal antibodies. These in addition to considering host factors such as the microbiome, which can affect medication metabolism and uptake, and tumor-associated molecular and immunological characteristics such as mutational burden, expression of checkpoint molecules and immune cell infiltration in the TME, may ultimately determine patient response to treatment and help optimize immunotherapy for melanoma.

## Author Contributions

DK, SK, KL, and SP conceived and designed the study; DK, HB, SK, BT, CB, ET, AS, NT, KL, and SP searched and studied the literature; DK, SK, BT, CB, ET, AS, NT, KL, and SP wrote the manuscript; DK, SK, HB, KL, and SP discussed and interpreted the literature findings; SM, SC, GP, AK, MN, RJH, EF, RMH, AC, IW, JS, DJ, JG, CH, and AM commented and helped to edit the manuscript; SK supervised the study.

### Conflict of Interest Statement

The authors declare that the research was conducted in the absence of any commercial or financial relationships that could be construed as a potential conflict of interest.
